# 1746. RSV seasonality patterns change during 2016 to 2020 among hospitalized young children in Taiwan

**DOI:** 10.1093/ofid/ofad500.1577

**Published:** 2023-11-27

**Authors:** Ching-Hu Chung, Szu-Ying Tsai, Ting-Yu Yeh

**Affiliations:** Department of Medicine, Mackay Medical College, New Taipei City, Taipei, Taiwan; Sanofi, Taipei, Taiwan, Taipei City, Taipei, Taiwan; Sanofi, Taipei, Taiwan, Taipei City, Taipei, Taiwan

## Abstract

**Background:**

Respiratory syncytial virus (RSV) is a common cause of hospitalizations in young children. Previous studies in Taiwan demonstrated seasonality of RSV hospitalization (RSVH) in young children was rather year-round with small peaks in spring and fall. This study aims to investigate recent seasonality patterns of RSVH in Taiwan using latest National Health Insurance (NHI) database.

**Methods:**

We linked Taiwan birth cohort database to the NHI database for the years 2016-2020. Children < 5 years having hospital admission record with RSV ICD-10 coding were defined as having RSVH. To assess the correlations between RSVH with monthly average temperature/humidity, we used data from 2016-2019 (not including 2020 due to unusual RSV endemic) and by geographic region (Northern, Central, and Southern Taiwan).

**Results:**

In children < 1 year, the annual RSVH incidence increased steadily from 1.32% in 2016, 1.55% in 2017, 1.60% in 2018, 1.67% in 2019, to 1.71% in 2020 (p=0.03). In contrast, for children aged 1-4 years, the annual RSVH incidence remained somewhat stable from 0.29%, 0.33%, 0.36%, 0.31% from 2016 to 2019, then substantially increased to 0.76% in 2020 (a 2.47-fold increase compared to 2019, p=0.22). Only one RSVH peak was observed per year for both age groups: in May for 2016, September for 2017-2019 and November for 2020. Temperature and RSVH showed high correlation among years 2016-2019, with correlation coefficients 0.64-0.68 and 0.64-0.66 in children aged < 1 and 1-4 years, respectively. However, RSVH only showed low to moderate correlation with humidity in both age groups.

**Conclusion:**

The previously established seasonality patterns of RSVH in Taiwan were disrupted. A higher RSVH incidence for children aged 1-4 years was noted during 2020 endemic than previous years. Only one RSVH peak was observed in 2016-2020, and its correlation with temperature was high.

**Disclosures:**

**Ching-Hu Chung, PhD**, Sanofi and AstraZeneca: Grant/Research Support **Szu-Ying Tsai, n/a**, Sanofi: employe **Ting-Yu Yeh, n/a**, Sanofi: employe

